# How Could COVID-19 Change Scholarly Communication to a New Normal in the Open Science Paradigm?

**DOI:** 10.1016/j.patter.2020.100191

**Published:** 2021-01-08

**Authors:** Kazuhiro Hayashi

**Affiliations:** 1 National Institute of Science and Technology Policy (NISTEP), Tokyo, Japan

## Abstract

Author reviews digital transformation of scholarly communication since 1990s and explains how COVID-19 is accelerating open science, with some analogy of chemical reactions. Discussing the current situation of preprint, the potential of peer review, and the essence of open science, developing additional services and balancing incremental and innovation in the transition state is crucial to foster new trust among stakeholders.

## Main Text

Since the emergence of the world wide web, scholarly journals have been transformed practically into “online journals” and have developed many useful functions such as online submission, search, and connecting to various items beyond just citation linking for better scholarly communication. It has been a while since online journals became inevitable among the researchers of various fields. Still, the framework of journal publishing itself, especially the scheme of peer review, has not been changed for many years, although it has been improved partially or additionally by the use of online reviewing systems. Some radical challenges such as open peer review have been tried by publishers and others, but these attempts have failed or only succeeded in a small range so far. The current framework of journal publishing and peer review is very robust, and it looks like it’s too established to change drastically.

**Figure 1:**
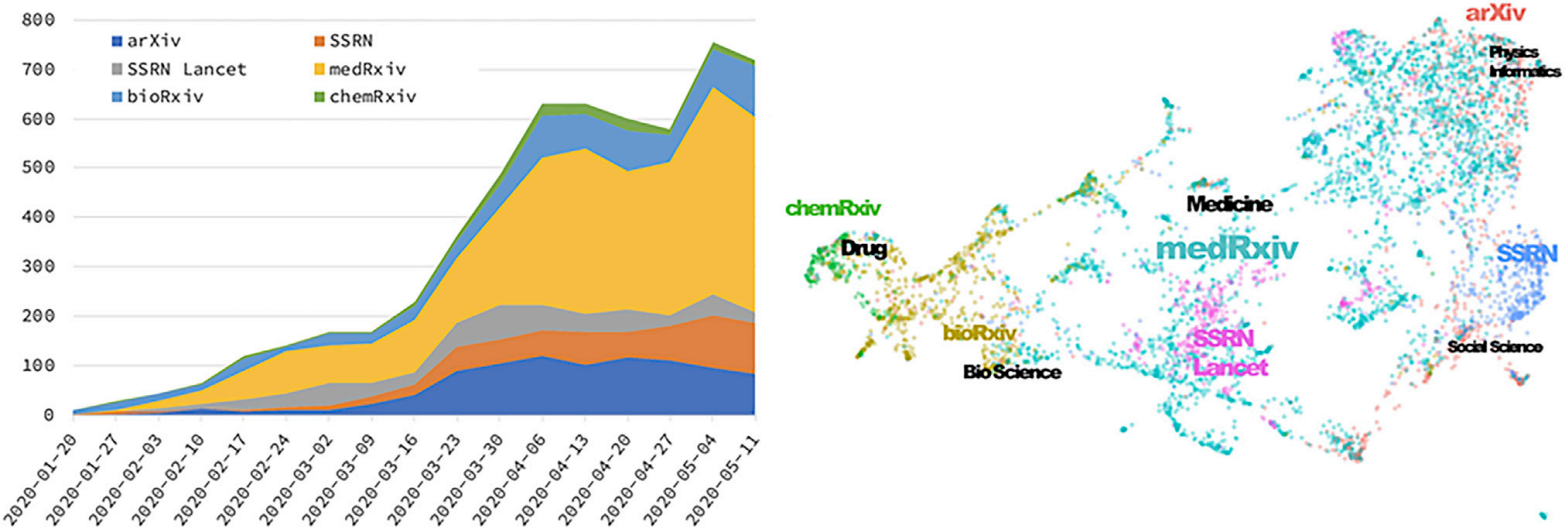
Rapid Growth of Preprint and Cluster Network of Preprints on Major Preprint Servers Regarding COVID-19

However, COVID-19 is now changing academic publishing dramatically—changing the whole academic society just like it is changing our whole society, including industry. COVID-19 research is accelerating open science more rapidly than ever before. For example, preprints are helping researchers share their research outputs immediately, sometimes along with their datasets. In addition, like a physicist trying to analyze the COVID-19 dataset and proposing a model, cross-disciplinary communication is happening regularly. Here, data science is playing a significant role. Furthermore, trans-disciplinary communication between scientists and citizens is also happening to solve this crisis. At this point, we must recognize that such emerging communications are happening but are not closely related to the current established journal publishing scheme. Current established scholarly communication seems to be too slow to fit current emerging communications on emergency situations such as a global pandemic. These open and rapid communications also affect the analysis of science and technology trends. Figure 1 is a preliminary cluster analysis of preprints regarding COVID-19 in various preprint servers.^1^ We already have a method to understand the research trend without analyzing in depth each article published.

**Figure 2:**
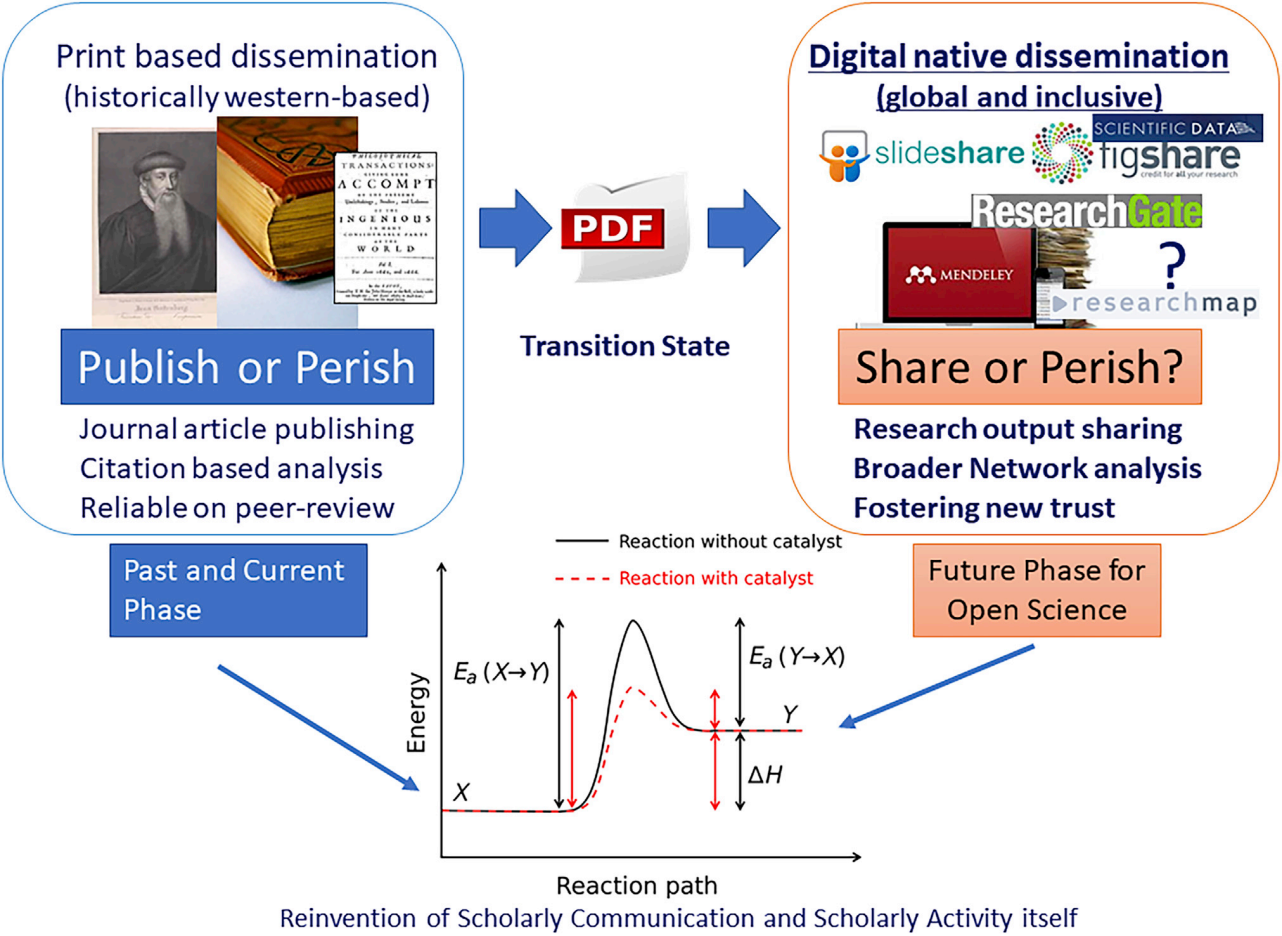
Paradigm Shifting toward Open Science

Since I majored in organic synthesis, I sometimes use the analogy of chemical reactions to explain the transformation of paradigm when I have a chance to explain open science to various stakeholders. One good example is using a concept of “activation energy” for paradigm shifting. Let’s recall the transformation of e-journals from paper journals. You may know or not, that publishers and learned societies consumed many resources (time, money, and human) and energy (including passion) to move forward, which was more than they assumed in the earlier stage. We need much more activation energy for paradigm shifting than it apparently needs. Beyond just e-journals, we are entering the digital native paradigm that open science is foreseeing. I often change the phrase “publish or perish” to “share or perish” to communicate the image of the open science paradigm (Figure 2). We are changing print-based dissemination to digital native dissemination, sharing research outputs (not just journal article publishing) and network analysis with data science for better research evaluation, in addition to citation-based analysis. However, the academic community was still skeptical even if it understood its potential fully. We needed much more energy to change the paradigm. Now, COVID-19 is providing us tons of energy to move forward.

What will happen practically? From my view with this current situation, the below points will become a custom among scientists in general, not just specifically by field.

1 Preprint first (at least for authors’ priority)
2 Data sharing as a common practice (like citations) and partially mandated (not all)
3 Filtering with multidimensional views, which comes through more transparent methods, keeping established editorship and peer review for a trusted authorization

These items are beneficial for both researchers and governments (funders). As far as I know, every policy development in open science or better research evaluation is keen on the rapid and transparent dissemination of publicly funded research outputs.

Furthermore, these new customs would urge us to make a progress of the issues listed below.

1 Re-framing or re-inventing peer review itself as a new digital native filtering process with preprints (how can we evaluate huge amounts of incoming preprints rapidly?)
2 Establishing curatorship like an advanced version of editorship within the new scheme of peer review (the editor role would change or expand to find good papers on the web and in the preprint servers)
3 Developing “contributorship” beyond authorship, especially for research data, and ultimately for the whole research process (as we recognize all the various contributors to research)

I should say most of these issues have been discussed many times and solutions have been fully or partially tested by some publishers or other initiatives. They have not been solved yet, as it is difficult to make these solutions common practice, despite the fact that the information and communication technology (ICT) for those schemes are ready to go. The things publishers and other publishing-related initiatives are now dealing with because of COVID-19, like expedited peer review, de facto open access, and data sharing, are things that they’ve been wanting to do in open dcience for a while. Then, we don’t have to create all the functions for open science from the beginning—we should gather, review, and arrange past challenges to redesign digital native scholarly communication within the current context strongly energized by COVID-19.

**Figure 3:**
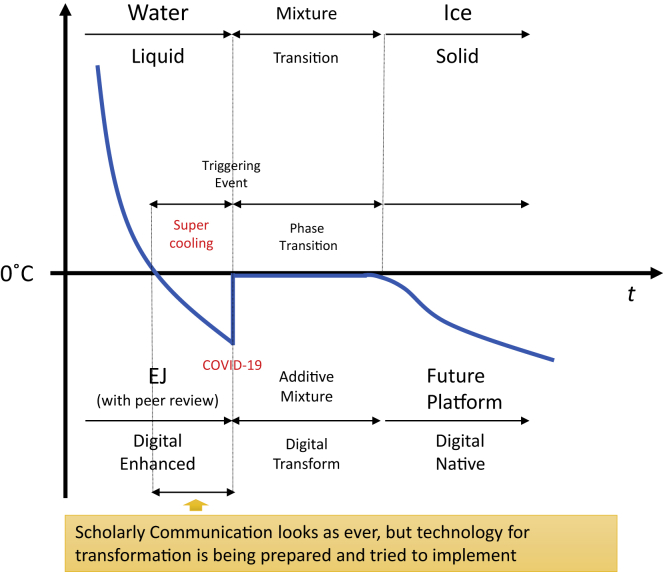
Phase Transfer from Water to Ice with Supercooling Time

I would like to introduce another chemical reaction to understand the current situation. We know that water normally freezes at 273.15 K (0°C, 32°F), but it can be “supercooled” to its crystal homogeneous nucleation (so-called ice) being kept as liquid (Figure 3). During the supercooling period, the water looks just like water, but the ice is crystalized instantly with a trigger event like vibration or just ticking. Like that, I explained many times that the ICT has been ready for many years to change the current framework of scholarly communication drastically. Scholarly communications have already passed a theoretical tipping point, but the practice has not changed, just like the state of supercooled water. We are just waiting for the trigger event to change the state—an event that no one could have predicted. I sometimes use this story to tell the movement of open access, which was originally initiated in 1990s. After many challenges had been tried in the 2000s, I believe the success of PLoS, especially PLoS ONE, triggered its phase transfer to let many established publishers launch OA journals in the 2010s.

And now COVID-19 is unexpectedly but definitely triggering the crystallization from the past supercooled liquid state to change the whole scholarly communication framework rapidly in the 2020s. Once the framework is shifted, we cannot go back because it has already passed the theoretical tipping point. OA journals are already a de facto media in scholarly communication. So we—researchers, authors, reviewers, editors, publishers and librarians together—should enjoy exploring the new possibilities of scholarly communication with a lot of the assets and knowledge we have gained in the supercooling period. COVID-19 is the short, sharp shock that allows us to crystallize our supercooled water and make that state change happen for better scholarly communication.

Nevertheless, one important point we learned from the history of digitization is that we cannot totally alter or abandon the current established scheme of scholarly publishing and peer review in a short time. It’s reasonable to think that we are going to have an “additional and vital layer of services” in the transition state for future scholarly communication. For example, the barriers to publishing preprint are so low that there would be a lot of faulty, biased, and misleading information out there. And it would be spread instantly if it is catchy, or if it confirms people’s biases. In addition, we don’t have any common methodology to evaluate huge numbers of preprints rapidly, except for applying the current scheme of (expedited) peer review afterward. Also, we should learn from the fact that some people in the 1990s dreamed of paperless online-only journals with HTML, but the whole publishing market practically selected print-based PDF files for online journals. The market does not accept just a new service but welcomes a “new and trusted” service. And again, I say we have been in the supercooling period to develop some disruptive media or platform, maybe with preprint or ambitiously with research data, while PDF is acquiring trust, as an incremental evolution, waiting for the trigger event. Therefore, the new developing layer for scholarly communications has been accumulated on the current established layer we have fostered over 350 years, starting from the beginning of print journal publishing. We should promote both incremental and disruptive change and also create a synergy for the rapid and healthier dissemination of scholarly communication with trust.

The essence of open science is not in the technology itself, but in the formation of new practices and cultures by changing human behavior. It needs time to change the culture. Looking toward a digitally native science, we have already entered an era where new services are created and new trust is earned by new challenges on a current reliable foundation. And after the supercooling time I mentioned, COVID-19 drastically broke with the conventions of the past and forcibly evoked a new world, which is definitely an activation energy to pass the tipping point of transition toward new paradigm. Given that we have finally entered the era of developing a full-fledged digital native service, keeping the essence of scholarly communication, especially on peer review, is crucial and it will still take time to transform after we develop a future service. Balancing convention and innovation is still important for each stakeholder in these times. And what I am convinced of, even at this stage, is that data science helps to widely and deeply improve our current situation incrementally, make something happen, and convince stakeholders to earn their trust with data.
